# Analyzing evolutionary game theory in epidemic management: A study on social distancing and mask-wearing strategies

**DOI:** 10.1371/journal.pone.0301915

**Published:** 2024-06-25

**Authors:** Khondoker Nazmoon Nabi, Murshed Ahmed Ovi, K. M. Ariful Kabir

**Affiliations:** Department of Mathematics, Bangladesh University of Engineering and Technology (BUET), Dhaka, Bangladesh; Texas A&M University College Station, UNITED STATES

## Abstract

When combating a respiratory disease outbreak, the effectiveness of protective measures hinges on spontaneous shifts in human behavior driven by risk perception and careful cost-benefit analysis. In this study, a novel concept has been introduced, integrating social distancing and mask-wearing strategies into a unified framework that combines evolutionary game theory with an extended classical epidemic model. To yield deeper insights into human decision-making during COVID-19, we integrate both the prevalent dilemma faced at the epidemic’s onset regarding mask-wearing and social distancing practices, along with a comprehensive cost-benefit analysis. We explore the often-overlooked aspect of effective mask adoption among undetected infectious individuals to evaluate the significance of source control. Both undetected and detected infectious individuals can significantly reduce the risk of infection for non-masked individuals by wearing effective facemasks. When the economical burden of mask usage becomes unsustainable in the community, promoting affordable and safe social distancing becomes vital in slowing the epidemic’s progress, allowing crucial time for public health preparedness. In contrast, as the indirect expenses associated with safe social distancing escalate, affordable and effective facemask usage could be a feasible option. In our analysis, it was observed that during periods of heightened infection risk, there is a noticeable surge in public interest and dedication to complying with social distancing measures. However, its impact diminishes beyond a certain disease transmission threshold, as this strategy cannot completely eliminate the disease burden in the community. Maximum public compliance with social distancing and mask-wearing strategies can be achieved when they are affordable for the community. While implementing both strategies together could ultimately reduce the epidemic’s effective reproduction number ($R_{e}$) to below one, countries still have the flexibility to prioritize either of them, easing strictness on the other based on their socio-economic conditions.

## Introduction

Over the last few decades, emerging and re-emerging epidemics of infectious diseases have posed new challenges and threats to human health communities with alarming regularity [1]. Different countries have adopted various strategies to control the transmission of COVID-19, based on disease severity. Some countries deployed stringent lockdown policies as a precipitous reaction to the pandemic. However, this strategy is associated with significant socio-economic costs, which could cripple a country’s economy [2]. To date, public health experts and policy makers are still trying to solve the puzzle of extracting the maximum benefits from lockdowns with minimum economic disruption [3]. Maintaining a trade-off between public health and the economy is often challenging for policy makers in any country. In the absence of any widely available effective vaccine against the disease, non-pharmaceutical interventions (NPIs) can play pivotal roles as effective infectious contact-reduction strategies [4–6]. However, the effectiveness of these intervention strategies largely depends on human behavior and the management of inherent socio-economic costs [7–9]. Social distancing was considered to be one of the effective contact-reduction strategies in earlier pandemics as well [10]. The practice of social distancing, or physical distancing, refers to limiting one’s movement as much as possible, avoiding congregated places and mass gatherings. One potential outcome of social distancing measures is that they could prevent surges in daily infection cases by slowing down the transmission rate. Aiming to impede the disease transmission rate, numerous countries deployed this intervention policy during the COVID-19 outbreak [11]. Consequently, they gained some extra time to improve their supply chain management of medical equipment, beds and other healthcare facilities. However, the long-term implementation of this strategy can lead to epidemic fatigue, public apathy, social rejection, increased impersonality and individualism, loss of a sense of community, and economic recession [12, 13]. This may lead people to violate social distancing guidelines [14], potentially paving the way for future epidemiological waves. Health officials urge the need to maintain a distance of at least six feet from others during any ongoing infectious disease outbreak [15]. Mask-wearing, a systematic practice grounded in epidemiological principles, is an effective measure for reducing infectious contact in airborne disease control. It reduces transmissibility per contact by limiting the spread of infected respiratory particles [4, 16].

In response to an epidemic outbreak, human behavior can significantly impact the progression dynamics of the epidemic, as it has been intricately linked with the transmission of respiratory diseases. In the face of an outbreak, individuals can play essential roles in mitigating the outbreak’s severity by adhering to protective masks and social distancing. Numerous theoretical works have already been conducted on evolutionary game theory to understand human behavioural changes in various widespread respiratory disease outbreak settings [17–21]. To curb any airborne disease transmission in the community, mild or successive human behavioral changes can have a noticeable impact. Social distancing can influence the outbreak pattern, such as outbreak size, duration of the outbreak, or even multiple waves with relatively shorter inter-epidemic periods [10]. In complex social dilemma situations, public willingness and behavioral feedback towards self-protective measures can play an active role in mitigating airborne disease transmission during an epidemic [9]. When making a decision about adopting any intervention strategy, individuals may consider the inherent cost as well as the benefits of the decision [22]. In addition, one may think about the disease prevalence in the community as well. In a game-theoretic review study, Chang et *al*. [23] showed that individual decision-making dynamics depends on the disease prevalence and socio-economic cost of adopting intervention strategies. Funk et *al*. [7] considers the information dynamics associated with social distancing in a network setting by prescribing a reduction in contacts based on proximity to infection. To shape policymaking towards the true betterment of any society, Arefin et *al*. [24] proposed a generalised conceptual framework of social efficiency deficit (SED) in the context of several social dilemmas. SED can be an informative parameter in understanding the impact of different intervention strategies and coming up with an optimal trade-off [25]. Kabir et *al*. [26] highlighted the comparative and absolute advantages of mask-wearing at both the individual and population levels.

Several scientific studies strongly suggest that social distancing and mask-wearing can be considered as effective contact-reduction strategies in any airborne disease outbreak setting. Siedner et *al*. [6] showed that statewide social distancing measures curtailed the US COVID-19 mean daily case growth rate by 0.9% (95% CI: −1.4% to −0.4%; *p* < 0.001) after one incubation period (i.e. 4 days), and reduced the COVID-19 induced mortality rate by 2.0% (95% CI: −3.0% to −0.9%; *p* < 0.001) after 7 days of implementation. Another study [5] highlighted the fact that tailored social distancing brought about a 29% reduction in COVID-19 incidence and a 35% reduction in COVID-19 associated mortality. To battle various respiratory pandemics, facemasks have been suggested as one of the effective intervention strategies since the 14th century. Wu [16] identified the cloth mask as an effective tool for personal protection, and his laudable work was followed to control the 1910 Manchurian plague. In a recent observational study, Wang et *al*. [27] claimed that facemasks were 79% effective in preventing the COVID-19 transmission when primary case and family contacts used facemasks prior to symptom onset. Richard et *al*. [28] investigated the effectiveness of wearing facemasks as a non-pharmaceutical intervention during lockdown periods (with or without them) using a mathematical modeling framework. They found that universal adoption of facemasks could result in a significant reduction in the effective reproduction number, $R_{e}$ of the COVID-19 pandemic. They recommended the immediate use of facemasks conveying the time-worthy message ‘my facemask protects you; your facemask protects me’, as numerous inhabitants in Western countries are reluctant to wear masks. Mask-wearing, similar to vaccinations, encounters resistance due to particular challenges. Highly efficacious masks, while offering substantial protection, often come with drawbacks such as elevated costs and physical discomfort, including hindered breathing. These factors contribute to reluctance among individuals to consistently use these protective masks, despite their proven effectiveness in mitigating disease transmission. On the other hand, wearing a mask confers benefits not only to the individual wearing it but also to those in their vicinity. Reluctance towards mask wearing or anti-mask attitudes have been continuously reported during the COVID-19 pandemic. Anti-mask sentiments during the pandemic were observed in various communities, influenced by concerns about being mistakenly perceived as threatening. Protests against mask-wearing have taken place in various locations around the world, occasionally leading to violent incidents. It is important to understand that these sentiments were not limited to any particular racial or ethnic group. Multiple studies suggest that attitudes toward mask-wearing are not exclusively linked to race but represent a broader social phenomenon [29, 30]. Due to negative attitudes, stigma and other geopolitical factors, promoting a universal mask-wearing policy has always been a challenging task [31]. A recent analysis suggests that the efficacy of law enforcement in promoting compliance with health guidelines during the COVID-19 pandemic varies significantly across different political systems, with autocratic countries showing higher efficiency [32]. Meng et al. [33] applied evolutionary game theory to investigate the evolution of vaccination strategies, highlighting the need to communicate the positive and negative consequences of infection and vaccination to shape behaviors that mitigate the final epidemic size. Another study [34] indicates that, in the long term, individuals either uniformly adopt or reject preventive measures. The study introduces an evolutionary game-theoretic framework to better capture the dynamic nature of how individuals adopt non-pharmaceutical interventions (NPIs), focusing explicitly on mask-wearing behavior and validating the model with accurate data. In another recent study [35], two distinct scenarios are elucidated. In the first scenario, the implementation of strict policies is observed when individuals prefer anti-epidemic measures, and the government has a balanced or surplus budget. Conversely, the second scenario emerges when individuals start showing a discernible preference for personal freedom, and the government exhibits a tendency to eschew the adoption of restrictive policies. The disappearance of the epidemic is contingent upon either the subcritical disease transmission rate or the strictness of non-pharmaceutical interventions (NPIs). After analyzing the situation, we have noticed some shortcomings and suggest integrating two types of preventive measures, such as wearing masks and maintaining social distance, into an Evolutionary Game Theory (EGT) model to investigate both the spread of the epidemic and the related behavioral patterns.

In this study, we introduce an extended version of the game-theoretic modeling approach for epidemic dynamics to understand the broad spectrum of human behavior and decision-making among individuals faced with different intervention choices during a widespread disease outbreak. This extension integrates both social distancing and mask-wearing intervention strategies. The main aim of this study is to investigate the impact of social distancing and mask-wearing as infectious contact-reduction strategies under social learning dynamics in mitigating the severity of COVID-19 in the community. We present the mathematical analysis of our model, including the stability analysis of the epidemic free equilibrium. A range of numerical simulations and phase portrait analyses has been performed on the model to understand the driving factors influencing human decision-making and behavioral changes in adopting self-protective measures during an outbreak scenario. To identify the key mechanisms controlling the effective reproduction number ($R_{e}$), a key epidemic threshold, our model underwent global sensitivity analysis.

## Materials and methods

### Epidemic dynamics

In the context of epidemic outbreaks, mask-wearing and social distancing emerge as effective non-pharmaceutical interventions. However, the efficacy of these measures is intrinsically linked to the degree of public adherence. Empirical observations have highlighted a heterogeneous compliance with these guidelines, a phenomenon prominently manifested during the COVID-19 pandemic. This disparity in behavioral response poses a formidable challenge in mitigating the transmission of SARS-CoV-2. Existing literature presents a spectrum of findings concerning the influence of governmental decrees on mask utilization. Notably, some studies suggest that the enactment of such mandates correlates with only nominal enhancements in compliance rates. The prevalence of undetected infections stands as a critical determinant in the propagation of pathogens. The initial phase of the COVID-19 pandemic serves as a quintessential example, wherein a substantial portion of infections eluded detection, thereby accelerating the spread of the virus. Analysis by the Centers for Disease Control and Prevention (CDC) [36] revealed that during the early stages of the pandemic, reported cases represented merely a fragment of the actual infection rates. This underscores the pivotal challenge posed by asymptomatic carriers in the transmission network of the disease. To understand these scenarios, we introduce a compartmental model $\left(SEI^{U}I^{D}R/S_{M}E_{M}I_{M}^{U}I_{M}^{D}R_{M}\right)$, which can be considered as an extension of well-known susceptible–exposed–infected–recovered (SEIR) model [37, 38]. Here, *S*, *S*_*M*_, *E*, *E*_*M*_, *I*^*U*^ $I_{M}^{U},I^{D},I_{M}^{D},R$ and *R*_*M*_ are the fractions of the population that are susceptible, masked susceptible, exposed, masked exposed, undetected infectious, undetected masked infectious, detected infectious, detected masked infectious, recovered, masked recovered respectively. Our framework makes several key assumptions described as follows:

- The susceptible individuals are divided into two categories based on their willingness to adopt mask wearing: those who voluntarily participate and those who are reluctant or exhibit an anti-mask attitude. This division is crucial for understanding the dynamics of mask usage in the context of an epidemic.
- Despite government strict mandates, the actual practice of wearing masks may not significantly increase. The success of the mask-wearing practice hinges on various factors. This assumption is based on existing literature [39] that has shown only modest effects of mandates on mask usage.
- A large proportion of infections remain undetected due to various factors such as mild or absent symptoms, limited awareness, or lack of testing. In our model, infected individuals are categorized into ‘detected’ and ‘undetected’ to account for this aspect.
- We assume similar transmission rates for both detected and undetected individuals. This is based on the premise that knowledge of infection status does not necessarily lead to immediate or significant changes in behavior that could affect transmission rates.
- Our model does not explicitly account for the economic and societal costs associated with quarantine measures. This decision acknowledges the existence of such costs but does not directly incorporate them into the model, aiming to balance realism and simplicity.
- The model assumes a uniform average recovery rate for both detected and undetected infected individuals. This simplification does not account for possible variations in recovery rates due to factors such as age, underlying health conditions, or access to healthcare.
- We avoid excessive complexity that might arise from numerous compartments and rapid transitions [40], thereby enhancing interpretability and computational feasibility.

In our model, susceptible individuals are divided into two categories based on their willingness to adopt mask wearing: those who voluntarily participate (*S*_*M*_) and those who are reluctant or exhibit an anti-mask attitude (*S*). Symptomatic patients are the main drivers in airborne disease transmission. According to some evidence-based studies [41, 42], there are risks of transmission from presymptomatic, paucisymptomatic, and asymptomatic individuals. To incorporate this in our model, we define the *I*^*U*^ cohort as the unmasked undetected population segment and $I_{M}^{U}$ as their masked counterparts. Effective transmission rate is denoted by *β*, which is a product of contact rate and probability of transmission given a contact. To model the social distancing adherence as an evolutionary behavior during the epidemic’s onset, we introduce the rate at which individuals prefer to engage in social distancing to prevent infection as *x*_*d*_(*t*). The rate at which exposed individuals get detected as infectious individuals (*I*^*D*^) is denoted as *τ*. We assume a uniform average recovery rate for both detected and undetected infectious individuals, which is denoted as *γ*. Wearing efficacious face coverings, both detected and undetected infectious individuals can significantly impede the disease transmission [43]. To put this source control mechanism into modeling, *q* has been considered that can be defined as the parameter that quantifies the degree to which others receive the protective benefits from mask-wearing by infectious individuals, which ranges between 0 and 1. To be more specific, *q* = 0 indicates the full protective benefit (complete risk reduction) and *q* = 1 indicates no benefit (no risk reduction) to the others. The efficacy of blocking virus particles inside a face covering or facemask is denoted by *η*.

**Fig 1:**
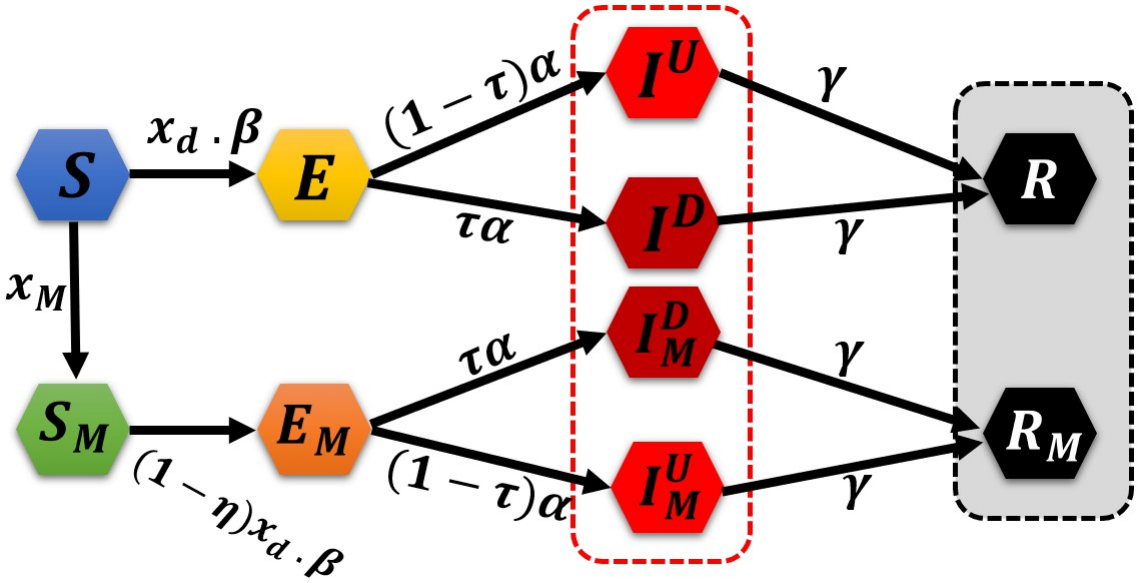
Schematic diagram of behavioral epidemiological model with social distancing and mask-wearing as infectious contact-reduction strategies. Both detected (*I*_*D*_) and undetected (*I*_*U*_) infectious individuals are considered. Proportion of mask-wearing individuals (*x*_*M*_((*t*))) and disease transmission reduction factor due to social distancing (*x*_*d*_(*t*)) have been modelled under evolutionary game dynamics.

The following system of differential equations (1) describes our proposed model and a schematic diagram describing the interactions of model cohorts is presented in Fig 1.

$$\begin{array}{c} \left\{ \begin{array}{cc} \frac{dS}{dt} & =-x_{d}\left(t\right)\beta S(I^{U}+I^{D}+q\left(I_{M}^{U}+I_{M}^{D}\right))-x_{M}\left(t\right)S,\\ \frac{dS_{M}}{dt} & =x_{M}\left(t\right)S-\left(1-\eta \right)x_{d}\left(t\right)\beta S_{M}(I^{U}+I^{D}+q\left(I_{M}^{U}+I_{M}^{D}\right)),\\ \frac{dE}{dt} & =x_{d}\left(t\right)\beta S(I^{U}+I^{D}+q\left(I_{M}^{U}+I_{M}^{D}\right))-\alpha E,\\ \frac{dE_{M}}{dt} & =\left(1-\eta \right)x_{d}\left(t\right)\beta S_{M}(I^{U}+I^{D}+q\left(I_{M}^{U}+I_{M}^{D}\right))-\alpha E_{M},\\ \frac{dI^{U}}{dt} & =\alpha \left(1-\tau \right)E-\gamma I^{U},\\ \frac{dI_{M}^{U}}{dt} & =\alpha \left(1-\tau \right)E_{M}-\gamma I_{M}^{U},\\ \frac{dI^{D}}{dt} & =\alpha \tau E-\gamma I^{D},\\ \frac{dI_{M}^{D}}{dt} & =\alpha \tau E_{M}-\gamma I_{M}^{D},\\ \frac{dR}{dt} & =\gamma I^{U}+\gamma I^{D},\\ \frac{dR_{M}}{dt} & =\gamma I_{M}^{U}+\gamma I_{M}^{D}. \end{array}\right. \end{array}$$ (1)

The parameters are described in Table 1.

**Table 1:** Definition of model parameters and their values used for numerical simulation.

Parameter	Description	Value	Source
*x*_*d*_(*t*)	rate at which individuals prefer to engage in social distancing to prevent infection	[0, 1]	(varied)
*β*	effective transmission rate	0.833	[44]
*q*	degree to which others receive the protective benefits from mask-wearing by infectious individuals	[0, 1]	(varied)
*x*_*M*_(*t*)	rate of wearing face coverings	[0, 1]	(varied)
*η*	efficacy of face coverings (benefit to them who wear face coverings)	[0, 1]	(varied)
*α*	incubation rate to be infectious	1/6	[8]
*τ*	rate of testing	[0, 1]	(varied)
*γ*	recovery rate	0.1	[22]
*d*	measure of social distancing (*d* = 0 indicates no distance maintained, while *d* = 1 signifies maintaining a safe distance from others)	[0, 1]	(varied)
*C*_*d*_	inherent socio-economic cost of maintaining social distancing	[0, 1]	(varied)
*C*_*D*_	relative socio-economic cost of maintaining social distancing	[0, 1]	(varied)
*C*_*m*_	perceived cost or inconvenience of using face coverings	[0, 1]	(varied)
*C*_*M*_	relative perceived cost or inconvenience of using face coverings	[0, 1]	(varied)
*C*_*i*_	cost associated with the risk of infection	[0, 1]	(varied)
*N*_0_	initial population size	1	(constant)
*M*	proportion of the population consistently wearing masks	[0, 1]	(varied)

### Behavioral model

Our behavioral model assumes that the players of the game are susceptible individuals who decide whether to follow contact reduction measures (compliance) or not (non-compliance) aimed at impeding the disease transmission. In case of adopting any strategy, the payoff can be calculated as a balance of the perceived pay-off of compliance (1 − *d*) ⋅ *C*_*d*_ against the payoff pertinent to getting infected through non-compliance *C*_*i*_ ⋅ *I*_*Total*_, where $I_{Total}=I^{U}+I_{M}^{U}+I^{D}+I_{M}^{D}$ refers to the total number of infectious people in an epidemic outbreak setting, and *d* refers to degree of practicing social distancing. When *I*_*Total*_ gets higher, people start getting panicked due to the rise of infection and start following safe social distancing. To be precise, *d* = 0 means no distance maintained and *d* = 1 means maintaining safe social distancing from others. For instance, maintaining at least 6 feet distance from others is suggested for COVID-19 [45]. *C*_*d*_ quantifies the “inherent socio-economic loss” for maintaining social distancing (ranging from 0 to 1), and *C*_*i*_ is the cost of infection. The term ‘pay-off’ denotes the socio-economic impact of not adhering to social distancing, expressed as (1 − *d*) ⋅ *C*_*d*_. If *d* = 0, indicating no social distancing, the pay-off would indeed be the full socio-economic impact (*C*_*d*_). The term (1 − *d*) ⋅ *C*_*d*_ does not imply a positive gain but reflects the balance or outcome of engaging or not engaging in social distancing. When *d* = 1 (complete adherence), the pay-off is 0, nullifying the socio-economic impact.

For any one of the behavioral decisions, the expected pay-off can be measured as [(1 − *d*) ⋅ *C*_*d*_ − *C*_*i*_ ⋅ *I*_*Total*_]. To facilitate analysis, we have rescaled the relative socio-economic cost as *C*_*D*_, where *C*_*D*_ = *C*_*d*_/*C*_*i*_, with *C*_*i*_ representing the infection cost, and it is standardized at *C*_*i*_ = 1.0. If *x*_*d*_(*t*) is the rate at which a specific proportion of people choose to maintain social distancing with a view to limiting the risk of getting infected at time *t*, then the rate at which individuals choose to maintain social distancing in an evolutionary mechanism can be described as follows:

$$\begin{array}{c} \frac{dx_{d}\left(t\right)}{dt}=m\cdot x_{d}\left(t\right)\cdot \left(1-x_{d}\left(t\right)\right)\left[\left(1-d\right)\cdot C_{D}-I_{Total}\right] \end{array}$$ (2)

where, m refers to proportionality constant which is converting the proportion of individuals into transmission rate.

In the current behavioral model under the evolutionary framework, individuals take decisions on whether to comply with mask wearing practice or not based on the cost of wearing efficacious face coverings and the cost of infection due to non-compliance. Hence, the payoff gain depends on the difference between the perceived payoff of wearing efficacious face coverings (−*M* ⋅ *C*_*m*_) and the cost associated with the risk of infection, resulting from non-compliance with mask-wearing guidelines (*C*_*i*_ ⋅ *I*_*Total*_). Here, *M* is defined as the fraction of the total population that consistently adopts the practice of wearing masks. This includes all individuals across different stages of the disease who have adopted mask-wearing, making $M=S_{M}+E_{M}+I_{M}^{U}+I_{M}^{D}+R_{M}$, where *S*_*M*_, *E*_*M*_, $I_{M}^{U}$, $I_{M}^{D}$, and *R*_*M*_ represent masked susceptible, masked exposed, masked undetected infected, masked detected infected, and masked recovered individuals respectively. As the value of *M* increases, indicating a higher number of masked individuals, there emerges a tendency for ‘free-riding‘, which describes a scenario where a portion of the population opts against mask usage and relies on masked individuals. This behavior becomes increasingly prevalent in the society as *M* becomes substantially large. To account for this inverse relationship, we incorporate a negative sign before *M* in the equation. *C*_*m*_ represents the perceived cost or inconvenience of mask-wearing, and $I_{Total}=I^{U}+I_{M}^{U}+I^{D}+I_{M}^{D}$ is the total number of infectious people in the population. For choosing any strategy, the expected payoff gain can be calculated as [−*M* ⋅ *C*_*m*_ + *C*_*i*_ ⋅ *I*_*Total*_]. After rescaling the above equation, the expected payoff becomes, [−*M* ⋅ *C*_*M*_ + *I*_*Total*_] in which *C*_*M*_ = *C*_*m*_/*C*_*i*_ and *C*_*i*_ = 1.0. The overall payoff gains in our model, therefore, depends on the balance between two factors: the cost of wearing a mask and the risk of getting infected. It is important to note that this model focuses on the perceived costs and benefits from the perspective of individuals making decisions about mask-wearing. If *x*_*M*_(*t*) represents the rate observed at time *t* at which susceptible people start wearing efficacious face coverings to protect themselves from airborne transmission as social distancing, then the time evolution of *x*_*M*_(*t*) can be described as follows:

$$\begin{array}{c} \frac{dx_{M}\left(t\right)}{dt}=m\cdot x_{M}\left(t\right)\cdot \left(1-x_{M}\left(t\right)\right)\left[-M\cdot C_{M}+I_{Total}\right] \end{array}$$ (3)

Eqs (2) and (3) represent the behavioral process in which the “third brackets” give the internal equilibrium for social distancing (*x*_*d*_(*t*)) and mask wearing (*x*_*M*_(*t*)) other than two trivial equilibria at *x*_*d*_(*t*)(*x*_*M*_(*t*)) = 0 and *x*_*d*_(*t*)(*x*_*M*_(*t*)) = 1. Based on the expected payoffs [(1 − *d*) ⋅ *C*_*D*_ − *I*_*Total*_] for social distancing and [−*M* ⋅ *C*_*M*_ + *I*_*Total*_] for the mask wearing, individuals switch their strategies. The payoff gains for switching strategies and its sign determines whether social distancing or mask-wearing is the favored switch. Suppose *ΔE* is the gain payoff between two strategies: *E*_*D*_, payoff to social distancing and *E*_*i*_, payoff to infected. To evaluate these two strategies, we assign an expected payoff to each strategy as, *E*_*D*_ = (1 − *d*) ⋅ *C*_*D*_ and *E*_*i*_ = −*I*_*Total*_ [18].

For our proposed game-theoretic model (1), average social payoff (ASP), a measure of overall situation of a society amid a disease outbreak, can be described as follows:

$$\begin{array}{c} \text{Average}\;\text{social}\;\text{payoff,}\;\text{ASP}=-S_{M}\left(\infty \right)C_{M}-BPD\left(\infty \right)C_{D}-FES\left(\infty \right) \end{array}$$ (4)

BPD is defined as the proportion of individuals who get benefits from physical distancing or social distancing practice during the COVID-19 epidemic. Firstly, we consider the behavioral dynamics in the absence of social distancing (ASD) practice (i.e. *x*_*d*_(*t*) = 1). We calculate the final epidemic size (FES) under conditions without social distancing (ASD), representing the total number of individuals who have experienced infection during the outbreak. This is denoted as FES_ASD_ at steady-state equilibrium. Afterwards, FES_PSD_ is calculated considering evolutionary mechanism in the presence of social distancing provision (PSD) (2), where the relationship between BPD and FES can be defined as follows:

$$\begin{array}{c} \text{BPD}={\text{FES}}_{\text{ASD}}-{\text{FES}}_{\text{PSD}} \end{array}$$ (5)

### Positivity and boundedness of solutions

The non-negativity of all state variables of our model (1) is shown in this section, i.e, solutions of the model (1) with positive initial data remain positive for all time *t* > 0. The following result can be obtained.

**Lemma 0.1** *Solutions of the game-theoretic model* (1) *having positive initial conditions remain positive for all t* ≥ 0.

*Proof*. Assume that $\phi \left(t\right)=(S\left(t\right),S_{M}\left(t\right),E\left(t\right),E_{M}\left(t\right),I^{U}\left(t\right),I_{M}^{U}\left(t\right),I^{D}\left(t\right),I_{M}^{D}\left(t\right),R\left(t\right),R_{M}\left(t\right))$ is a solution of (1) with positive initial conditions. Let us consider *E*(*t*) for *t* ≥ 0. From the third equation of the system (1) we get that,

$$E\left(t\right)=E\left(0\right)\;\text{exp}\;[\int_{0}^{t}(x_{d}\left(t\right)\beta S\left(t\right)(I^{U}\left(t\right)+I^{D}\left(t\right)+q\left(I_{M}^{U}\left(t\right)+I_{M}^{D}\left(t\right)\right))-\alpha E\left(\tau \right))d\tau ].$$

Since *E*(0) ≥ 0, it follows that *E*(*t*) ≥ 0 for *t* ≥ 0. The same procedure can be followed for *S*(*t*), *S*_*M*_(*t*), *E*_*M*_(*t*), *I*^*U*^(*t*), $I_{M}^{U}\left(t\right)$, *I*^*D*^(*t*), $I_{M}^{D}\left(t\right)$, *R*(*t*) and *R*_*M*_(*t*).

**Lemma 0.2** *Solutions of the model* (1) *having positive initial conditions are bounded by the total population i.e. N*_0_.

*Proof*. Let $S\left(t\right)+S_{M}\left(t\right)+E\left(t\right)+E_{M}\left(t\right)+I^{U}\left(t\right)+I_{M}^{U}\left(t\right)+I^{D}\left(t\right)+I_{M}^{D}\left(t\right)+R\left(t\right)+R_{M}\left(t\right)=N_{0}$. Thus, in absence of disease, we have

$$\frac{dN(t)}{dt}=0\Leftrightarrow N\left(t\right)=N_{0}$$

where *N*_0_ is equal to initial size of the total population. It follows that for all *t* ≥ 0, we have *S*(*t*) ≤ *N*_0_, *S*_*M*_(*t*) ≤ *N*_0_, *E*(*t*) ≤ *N*_0_, *E*_*M*_(*t*) ≤ *N*_0_, *I*^*U*^(*t*) ≤ *N*_0_, $I_{M}^{U}\left(t\right)\leq N_{0}$, *I*^*D*^(*t*) ≤ *N*_0_, $I_{M}^{D}\left(t\right)\leq N_{0}$, *R*(*t*) ≤ *N*_0_ and *D*(*t*) ≤ *N*_0_ with *N*(*t*) = *S*(*t*) + *E*_1_(*t*) + *E*_2_(*t*) + *I*(*t*) + *A*(*t*) + *Q*(*t*) + *L*(*t*) + *R*(*t*) + *R*_*M*_(*t*) = *N*_0_.

In what follows, we study the model (1) in the following set

$$\begin{array}{c} \begin{array}{cc} D & =\{(S\left(t\right),S_{M}\left(t\right),E\left(t\right),E_{M}\left(t\right),I^{U}\left(t\right),I_{M}^{U}\left(t\right),I^{D}\left(t\right),I_{M}^{D}\left(t\right),R\left(t\right),R_{M}\left(t\right){)}^{\prime }\in R_{+}^{10}:\\ & S\left(t\right)+S_{M}\left(t\right)+E\left(t\right)+E_{M}\left(t\right)+I^{U}\left(t\right)+I_{M}^{U}\left(t\right)+I^{D}\left(t\right)+I_{M}^{D}+R+R_{M}=N_{0}\} \end{array} \end{array}$$ (6)

which is positively-invariant and attracting region for the model (1).

### Asymptotic stability of disease-free equilibria

The disease-free equilibrium point denoted by $E_{0}$ can be defined as follows:

$$E_{0}=(S^{*},S_{M}^{*},E^{*},E_{M}^{*},I^{U*},I_{M}^{U*},I^{D*},I_{M}^{D*},R^{*},R_{M}^{*})$$

$$=(S^{*},S_{M}^{*},0,0,0,0,0,0,0,0)$$

where R* and $R_{M}^{*}$ can vary over the range [0, *N*_0_), S* and $S_{M}^{*}$ vary over [0, *N*_0_] and *N*_0_ represents the initial size of the total population. Matrices *F* and *V* for the new infection terms and the remaining transfer terms can be constructed as follows following the notations of Driessche and Watmough [46]. In our analysis, we calculate the time-dependent effective reproduction number, which is influenced by two parameters, *x*_*d*_ and *x*_*M*_, in distinct ways. Firstly, *x*_*d*_ represents the adherence parameter for maintaining social distancing, and it is entirely contingent on the dynamics of another game, making it a time-dependent factor. Secondly, Eq (12) illustrates the presence of two time-dependent compartments, denoted as *S*(*t*) and *S*_*M*_(*t*). This dual-compartment structure arises as a consequence of the parameter values *x*_*M*_. As *x*_*M*_ increases, the proportion of individuals in the *S*_*M*_ compartment also shoots up, while the fraction of population in the *S* compartment gets reduced simultaneously. The reason behind this shift is the transition from the non-masked (*S*) compartment to the masked (*S*_*M*_) compartment.

$$\begin{array}{c} \begin{array}{ccc} F & = & (\begin{array}{cccccc} 0 & 0 & x_{d}\left(t\right)\beta & qx_{d}\left(t\right)\beta & x_{d}\left(t\right)\beta & qx_{d}\left(t\right)\beta \\ 0 & 0 & \left(1-\eta \right)x_{d}\left(t\right)\beta & \left(1-\eta \right)qx_{d}\left(t\right)\beta & \left(1-\eta \right)x_{d}\left(t\right)\beta & \left(1-\eta \right)qx_{d}\left(t\right)\beta \\ 0 & 0 & 0 & 0 & 0 & 0\\ 0 & 0 & 0 & 0 & 0 & 0\\ 0 & 0 & 0 & 0 & 0 & 0\\ 0 & 0 & 0 & 0 & 0 & 0 \end{array}),\\ V & = & (\begin{array}{cccccc} \alpha & 0 & 0 & 0 & 0 & 0\\ 0 & \alpha & 0 & 0 & 0 & 0\\ -\alpha (1-\tau ) & 0 & \gamma & 0 & 0 & 0\\ 0 & -\alpha (1-\tau ) & 0 & \gamma & 0 & 0\\ -\alpha \tau & 0 & 0 & 0 & \gamma & 0\\ 0 & -\alpha \tau & 0 & 0 & 0 & \gamma \end{array}). \end{array} \end{array}$$ (7)

Then, the control reproduction number ($R_{c}$) is calculated as the spectral radius of the next generation matrix, *FV*^−1^ [46, 47]. For simplicity, *x*_*d*_ has been considered as a constant parameter.

$$R_{c}=\rho \left(FV^{-1}\right)=R_{I^{U}}+R_{I_{M}^{U}}+R_{I^{D}}+R_{I_{M}^{D}}=\frac{x_{d}\beta [1+q(1-\eta )]}{\gamma }$$ (8)

where *ρ*(⋅) refers to the spectral radius of the F-V matrix.

An informative epidemic threshold, control reproduction number ($R_{c}$) refers to the average number of new secondary cases generated by an infectious individual in a fully susceptible population where a certain portion is compliant with efficacious mask-wearing [48]. The following result can be claimed as a direct consequence of the next generation operator method [46, Theorem 2].

**Theorem 0.1**. *The airborne disease transmission dynamics is influenced by the control reproduction number* $R_{c}$ *as follows*:

1. *If* $R_{c}<1$ , *then the epidemic size rises exponentially to a peak and eventually dies out, i.e the disease-free equilibrium* $E_{0}$ *is locally asymptotically stable*.
2. If $R_{c}>1$, *then the epidemic size will generate a disease outbreak which means that the disease-free equilibrium* $E_{0}$ *is unstable*.

**Lemma 0.3**. *If* $R_{c}<1$, *then the disease-free equilibrium*, $E_{0}$ *is locally asymptotically stable and unstable if* $R_{c}>1$.

From a mathematical perspective, while lowering the $R_{c}$ to below 1 is necessary to eradicate the virus, the action alone might not be enough due to the existence of a potential of a stable endemic equilibrium. To ensure that the $R_{c}$ does not depend on the initial sizes of different sub-populations of the model, it is critical to show that the DFE is globally asymptotically stable (GAS), which establishes the local stability of the DFE, $E_{0}$ [49]. For $R_{c}$, the stability analysis is inconclusive. In this context, we claim the following result.

**Theorem 0.2**. *If* $R_{c}<1$, *then the manifold*, W, *of disease-free equilibrium points of the model* (1) *is GAS in* D.

The following Lyapunov function is considered to study the global stability of $E_{0}$.

$$\begin{array}{c} L=a_{1}E+a_{2}E_{M}+a_{3}I^{U}+a_{4}I_{M}^{U}+a_{5}I^{D}+a_{6}I_{M}^{D}. \end{array}$$ (9)

By deriving this function along the trajectories of the system (1), we obtain

$$\begin{array}{c} \begin{array}{cc} \dot{L} & =\;a_{1}\dot{E}+a_{2}{\dot{E}}_{M}+a_{3}{\dot{I}}^{U}+a_{4}{\dot{I}}_{M}^{U}+a_{5}{\dot{I}}^{D}+a_{6}{\dot{I}}_{M}^{D}\\ & =\;a_{1}[x_{d}\left(t\right)\beta S(I^{U}+I^{D}+q\left(I_{M}^{U}+I_{M}^{D}\right))-\alpha E]\\ & \;+a_{2}[\left(1-\eta \right)x_{d}\left(t\right)\beta S_{M}(I^{U}+I^{D}+q\left(I_{M}^{U}+I_{M}^{D}\right))-\alpha E_{M}]\\ & \;+a_{3}[\alpha \left(1-\tau \right)E-\gamma I^{U}]\\ & \;+a_{4}[\alpha \left(1-\tau \right)E_{M}-\gamma I_{M}^{U}]+a_{5}[\alpha \tau E-\gamma I^{D}]+a_{6}[\alpha \tau E_{M}-\gamma I_{M}^{D}] \end{array} \end{array}$$ (10)

We choose *a*_*i*_, *i* = 1, 2, …, 6, such that coefficients of *E*, *E*_*M*_, *I*^*U*^, $I_{M}^{U}$, *I*^*D*^, and $I_{M}^{D}$ become zero. That is, setting *a*_1_ = 1, *a*_2_ = *q* and *a*_3_ = 1, we finally obtain

$$\dot{L}\leq (R_{c}-1)E.$$ (11)

From (11), it follows that $\dot{L}<0$ if $R_{c}<1$, and $\dot{L}=0$ if and only if $E=E_{M}=I^{U}=I_{M}^{U}=I^{D}=I_{M}^{D}=0$. Therefore, L is a Lyapunov function for system (1). Moreover, the maximal invariant set contained in $\left\{\left(S\left(t\right),S_{M}\left(t\right),E\left(t\right),E_{M}\left(t\right),I^{U}\left(t\right),I_{M}^{U}\left(t\right),I^{D}\left(t\right),I_{M}^{D}\left(t\right),R\left(t\right),R_{M}\left(t\right)\right)\in \Omega :\dot{L}=0\right\}$ is the continuum of the disease-free equilibrium $\left(E_{0}\right)$. Hence, considering Lyapunov theory, the conclusion can be stated that disease-free equilibrium $\left(E_{0}\right)$ is GAS if $R_{c}<1$. Hence, it follows, by the LaSalle’s Invariance Principal, that the continuum of disease-free equilibria of the model (1) is a stable global attractor in Ω whenever $R_{c}\leq 1$. The previous analysis can be summarized as follows:

**Theorem 0.3**. *If* $R_{c}\leq 1$, *then the disease-free equilibrium* $E_{0}$ *is globally asymptotically stable on* Ω.

### Effective reproduction number

Another useful threshold quantity in epidemiology is the time-varying effective reproduction number, denoted by $R_{e}$. For the model (1),

$$\begin{array}{c} R_{e}=\frac{x_{d}\left(t\right)\beta \left[S^{*}\left(t\right)+q\left(1-\eta \right)S_{M}^{*}\left(t\right)\right]}{\gamma }. \end{array}$$ (12)

where, S*(*t*) represents the proportion of susceptible people who are non-adherent to mask-wearing and $S_{M}^{*}\left(t\right)$ represents the proportion of susceptible people who are adherent to mask-wearing. The number of disease cases rises when $R_{e}>1$, attains a peak when $R_{e}=1$, and declines when $R_{e}<1$ [48]. Fig 6 depicts an illustration of the effective reproduction number for our model (1).

## Results and discussion

### Sensitivity analysis

**Fig 2:**
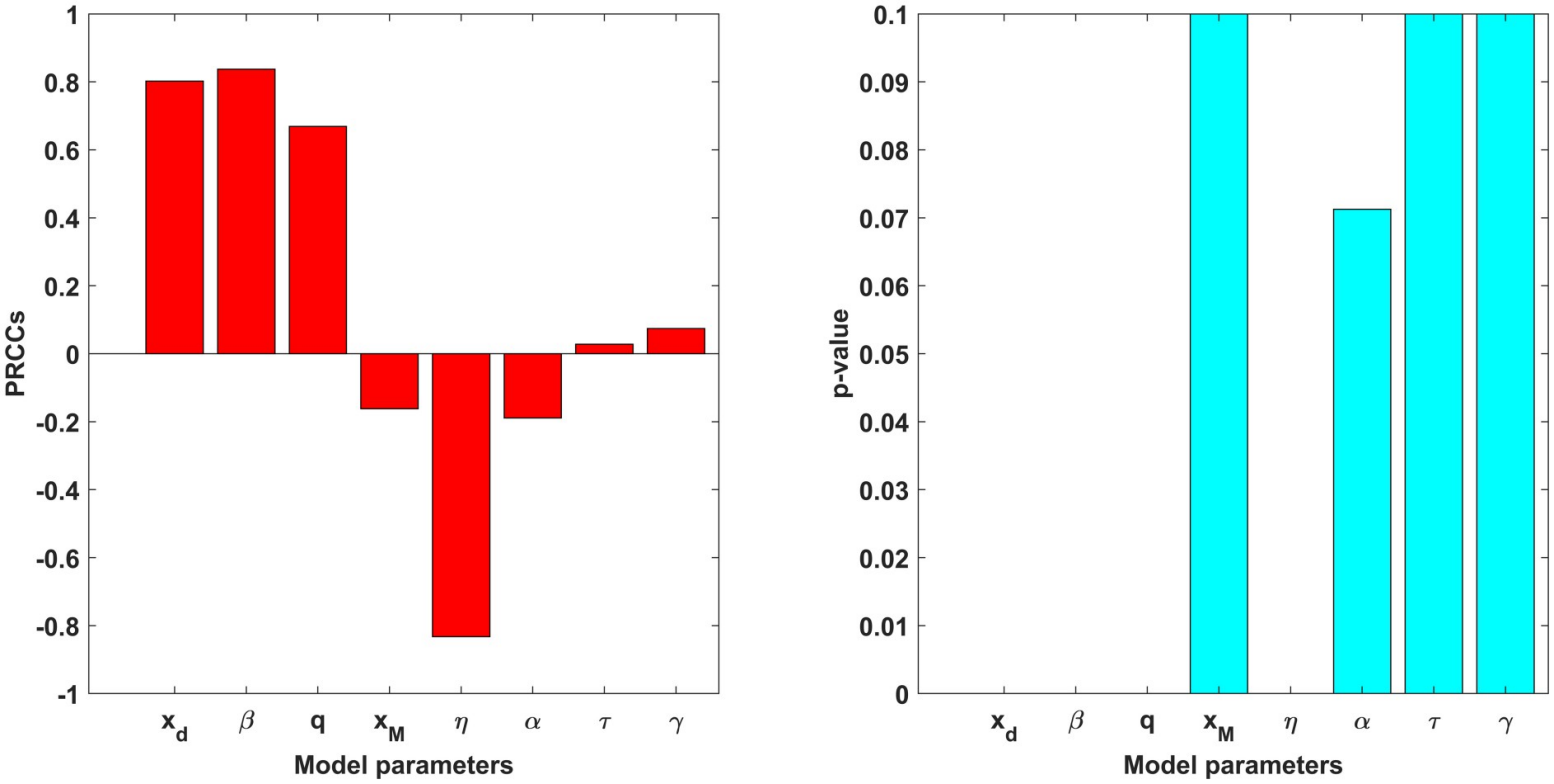
Partial rank correlation coefficients (PRCCs) of the effective reproduction number, $\left(R_{e}\right)$ of the model (1) with respect to the variations of the model (1) parameters. The baseline values of the parameters are considered from Table 1. The PRCC indices for most sensitive parameters: *η*, *β*, *x*_*d*_(*t*) and *q* are −0.847, 0.834, 0.82 and 0.67 respectively.

The epidemiological model (1) contains 11 parameters. Although some parameters are reported using historical airborne disease data, uncertainties are expected to arise in rest of the parameters of the model. Thus, it is crucial to measure the impact of the sensitivities of the parameters as an outcome of the numerical simulation results (for a particular response function, ($R_{e}$)). With an aim to determine the parameters of the model that have the most effect on the chosen response function, uncertainty and sensitivity analysis has been applied using Latin Hypercube Sampling technique and partial rank correlation coefficients (PRCCs) [50, 51]. The process of carrying out the sensitivity analysis involves defining a range (lower and upper bound) and distribution for each parameter of the model, and then splitting each parameter range into 1,000 equal sub-intervals. Without having any replacement, parameter sets are drawn from this space and a 1, 000 × 8 matrix is formed. Taking each row of this matrix into account to compute $R_{e}$ of the model and respective PRCCs. To assess the individual parameter contribution to the uncertainty and variability in $R_{e}$. Parameters with high PRCC values close to -1 or +1 are said to be highly correlated with the response function (those with negative (positive) PRCC values are said to be negatively (positively) correlated with the response function). We assume, for simplicity, that each of the 8 parameters (*x*_*d*_(*t*), *β*, *q*, *x*_*M*_(*t*), *η*, *α*, *τ* and *γ*) of the model (1) which are appeared at formula of $R_{e}$, obeys a uniform distribution. The range for each parameter is obtained by taking a deviation of 20% from its baseline value (given in Table 1). Our findings show that four parameters, namely the efficacy of face coverings at blocking both inward and outward transmission (*η*), effective contact rate (*β*), rate of reduced disease transmission due to social distancing (*x*_*d*_(*t*)), and mask-wearing benefits at population level (*q*) significant affect the response function ($R_{e}$) illustrated in Fig 2.

**Fig 3:**
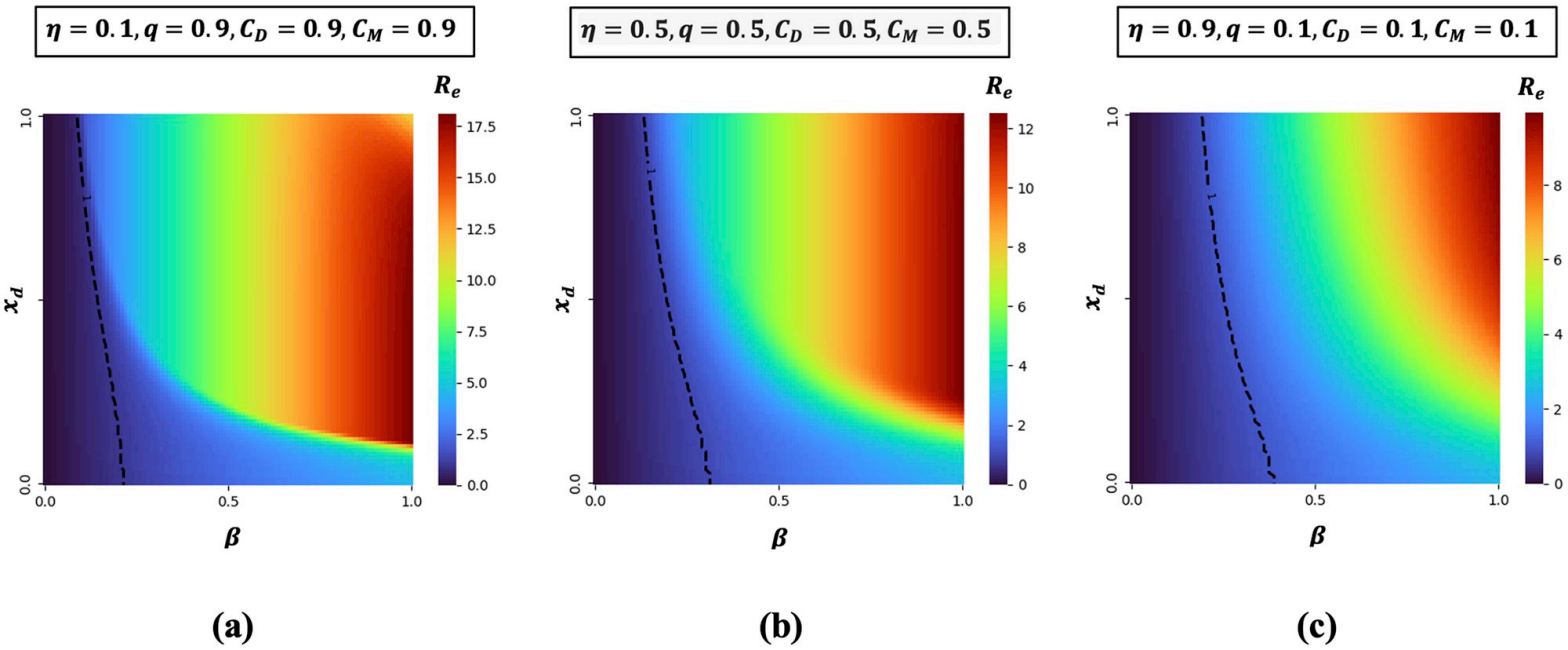
Heat maps of effective reproduction number ($R_{e}$) along two most sensitive parameters: effective transmission rate (*β*) and rate at which individuals prefer to engage in social distancing to prevent infection (*x*_*d*_(*t*)) for A: an exorbitant cost setting for both masks and social distancing with low mask efficacy and high (*η* = 0.1, *q* = 0.9, *C*_*M*_ = 0.9 and *C*_*D*_ = 0.9) B: *η* = 0.5, *q* = 0.5, *C*_*M*_ = 0.5 and *C*_*D*_ = 0.5 (a moderate cost setting for both masks and social distancing) and C: *η* = 0.9, *q* = 0.1, *C*_*M*_ = 0.1 and *C*_*D*_ = 0.1 (an affordable setting for both masks and social distancing). All remaining parameters remain constant and are derived from the values listed in the Table 1.

As stated above, the most imperative characteristic of the control reproduction number ($R_{c}$) is that it someway suggests the stability of the disease-free equilibrium. According to Eq (12), we consider effective reproduction numbers that act as a controlling function when we demonstrated for *β* versus *x*_*d*_(*t*) for three extreme situations displayed in Fig 3. Notably, the results drawn in Fig 3 illustrate that mass people could be encouraged effectively in adopting these contact-reduction measures if low socio-economic cost is ensured. Otherwise, a high disease burden could be witnessed by the mass people. The public health implication of these findings is general adherence to wearing a highly efficacious mask and tailored social distancing could bring ($R_{e}$) under one, reducing the number of detected and undetected infectious individuals. If the degree to which others receive the protective benefits from mask-wearing by infectious individuals is maximized, rapid disease transmission could be halted. Through their prosocial behavior, infectious carriers can play an impactful role in preventing the outward transmission of the virus wearing efficacious facemasks.

### Time series analysis

This section presents a time series analysis of our proposed classic compartmental epidemic model combined with human behaviour dynamics under EGT framework. For numerical solutions of the model (1), discretized finite difference method is used. Initially, we presumed the initial values as, *S*(0) ≈ 1.0, *x*_*d*_(0) ≈ 1.0 and *x*_*M*_(0) ≈ 0. In this study, we used *x*_*d*_(0) = 0.99 (when people start showing less preference to social distancing) [45] and *x*_*M*_(0) = 0.00001 (For simplicity, we set the initial mask-wearing rate as small as possible, which refers to minimum adherence to mask wearing practice) [26]. In addition, $S_{M}\left(0\right)=0,E\left(0\right)=0,E_{M}\left(0\right)=0,I^{U}\left(0\right)\approx 0,I_{M}^{U}\left(0\right)\approx 0,I^{D}\left(0\right)=0,I_{M}^{D}\left(0\right)=0,R\left(0\right)=0$ and *R*_*M*_(0) = 0. Throughout, we consider the same time scale known as local time scale (single season) for both epidemic disease spreading and strategy updating for mask wearing and social distancing [18, 19, 26, 44]. The baseline system values, defined as the default, for each of the parameters are taken from Table 1.

**Fig 4:**
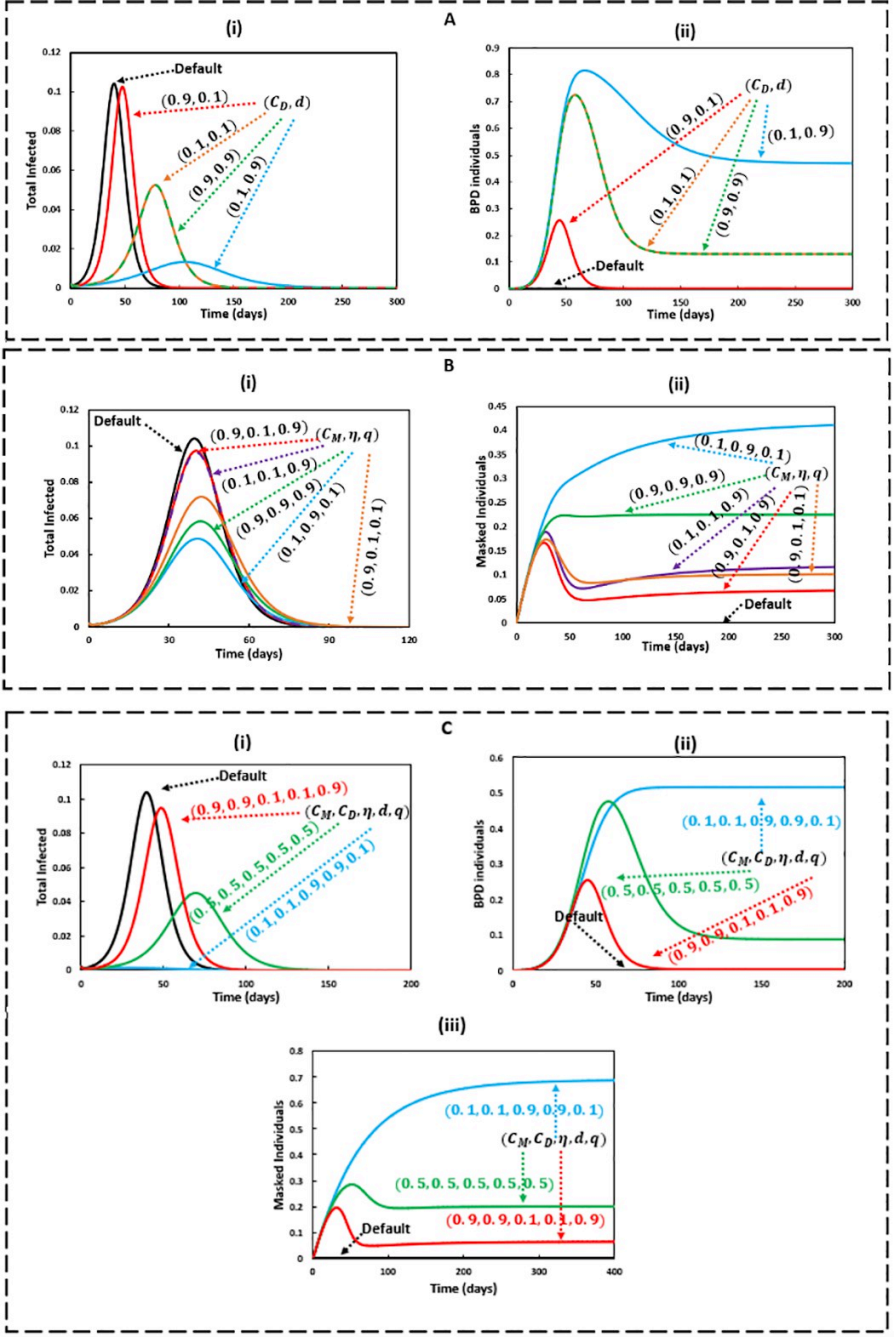
Panel A: time-series of (i) total infected and (ii) benefitted from social distancing (BPD) individuals when only social distancing is considered with variations of *C*_*D*_ and d. Panel B: time-series of (i) total infected and (ii) mask-wearing individuals when only mask-wearing is considered with variations of *C*_*M*_, *η* and *q*. Panel C: time-series of (i) total infected (ii) BPD individuals and (iii) mask-wearing individuals when both social distancing and mask-wearing are considered with variations of *C*_*M*_, *C*_*D*_, *η*, *d* and *q*.

In Fig 4, Panel A (i and ii) illustrates the fraction of total infected individuals and the fraction of individuals benefitted from social distancing (BPD) over time in case of only social distancing intervention strategy. In the absence of social learning dynamics (default-colored black), the worst situation regarding total infected cases is visible in the Panel A-i, which is approximately same as poor compliance with social distancing in an expensive relative socio-economic cost setting (for *C*_*D*_ = 0.9 and *d* = 0.1). Nevertheless, in an affordable relative cost setting (for *C*_*D*_ = 0.1 and *d* = 0.9), a significant reduction in total infected cases could be observed in the community, as most of the people pick out safe social distancing due to its cost-effectiveness. Our findings highlight the fact that social distancing can flatten the curve and can be treated as an effective tool of delaying epidemic peak. However, inaffordable social distancing cost can exacerbate the disease outbreak situation. Although social-learning dynamics generates different scenarios, all settings eventually reach a stable equilibrium. When the socio-economic cost of maintaining safe social distancing (*d* = 0.9) reaches rock-bottom (*C*_*D*_ = 0.1) illustrated in Panel A-ii, a significant fraction of individuals get benefitted from this intervention strategy compared to other settings.

Wearing-mask is another effective contact-reduction strategy in controlling a disease outbreak illustrated in Panel B (Fig 4). Panel B (ii) highlights that high facemask compliance could be observed among mass people if highly efficacious facemasks (*η* = 0.9) are available at cheap price (*C*_*M*_ = 0.1) and maximum mask benefits to the community (*q* = 0.1) is ensured. Importantly, this intervention strategy can be used to flatten the curve. However, this strategy has failed to delay the peak occurrence.

The combination of the two aforementioned contact-reduction policies could significantly avert an airborne disease outbreak in a low cost setting depicted in Panel C (Fig 4). The widespread adoption of contact-reduction strategies can be achieved by providing effective facemasks at affordable prices and minimizing the socioeconomic costs associated with maintaining physical distancing, thus motivating a larger portion of the population to embrace these measures illustrated in Panel C (ii and iii).

### Phase portrait analysis

**Fig 5:**
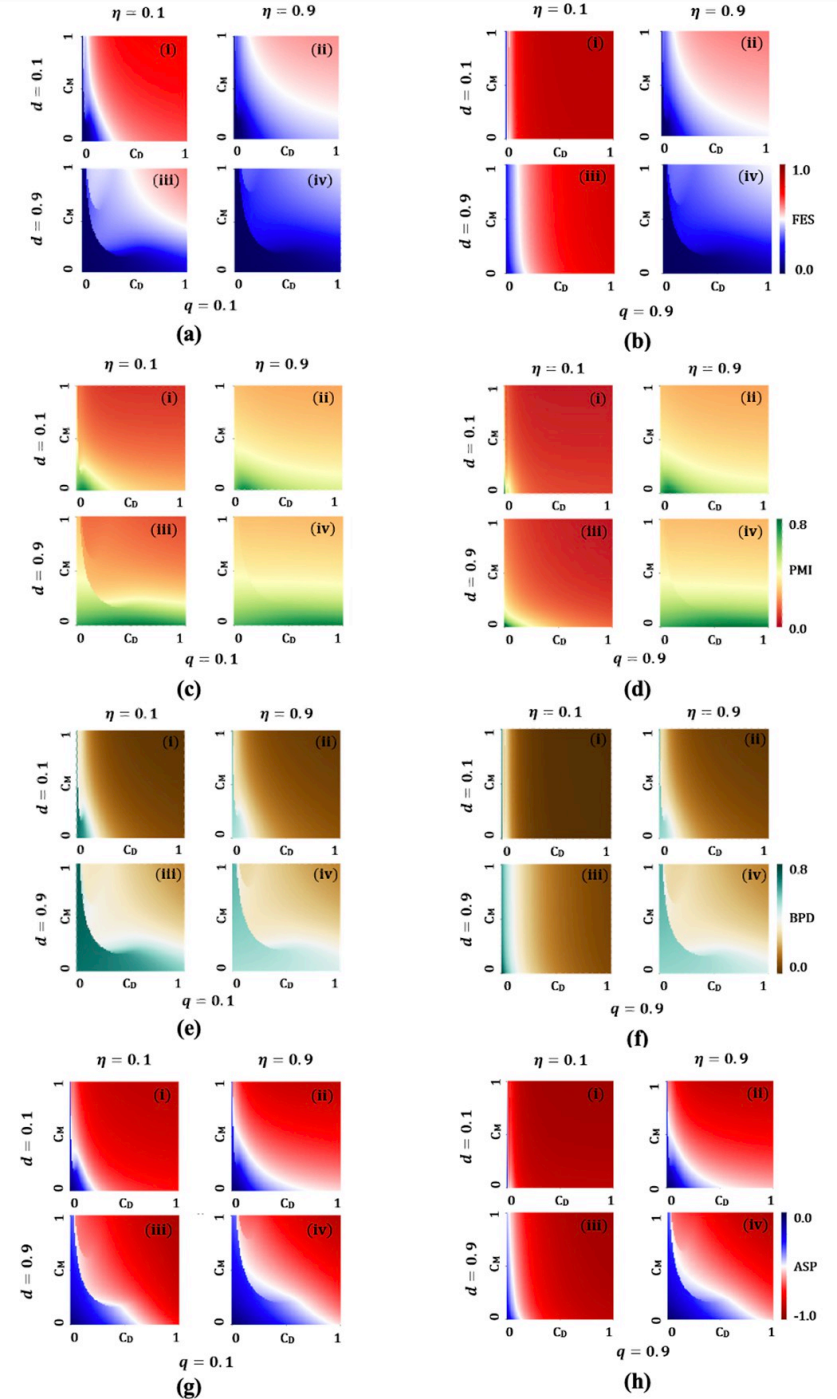
Two-dimensional phase diagram of ((a)-(b)) final epidemic size (FES), ((c)-(d)) proportion of masked individuals (PMI), ((e)-(f)) BPD individuals and ((g)-(h)) average social payoff (ASP), for parameter space *η* = 0.1 and 0.9 and *d* = 0.1 and 0.9 and *q* = 0.1 and 0.9 along the socio-economic cost of social distancing, *C*_*D*_, and the economic cost of wearing mask, *C*_*M*_. To construct the phase portraits, plausible parameter values from Table 1 are considered.

To gain valuable insights into how varying the cost of face coverings (*C*_*M*_) and relative cost of social distancing (*C*_*D*_) affect the final epidemic size (FES) (Fig 5a and 5b), proportion of masked individuals (PMI) (Fig 5c and 5d), proportion of individuals benefitted from social or physical distancing (BPD) (Fig 5e and 5f) and average social payoff (ASP) (Fig 5g and 5h), a collection of 2D heat maps are displayed in Fig 5. Explicitly, each block (i), (ii), (iii) and (iv) illustrate different settings of mask efficacy and distancing parameters (*η*, *d*) as (0.1, 0.1), (0.9, 0.1), (0.1, 0.9) and (0.9, 0.9), respectively, in which left-side Fig 5a, 5c, 5e and 5g and right-side Fig 5b, 5d, 5f and 5h present the impact of different degrees protective benefits received by others from mask-wearing by infectious individuals, *q* = 0.1 (left) and *q* = 0.9 (right).

From Fig 5a and 5b, as the values of both *C*_*M*_ and *C*_*D*_ increase, FES starts mounting as expected, since individuals are less likely to wear efficacious face coverings and inadherent to maintain social distancing in a relatively high-cost settings. Moreover, when people start maintaining safe social distancing from others (*d* = 0.9) and practice wearing highly efficacious face coverings (*η* = 0.9) simultaneously, the disease burden can be mitigated comprehensively. A comparison of Fig 5a and 5b highlights the fact that a mask wearer ensuring maximum mask benefits to his or her neighbors (*q* = 0.1) in Fig 5a, can play a significant role in attenuating the disease burden in the community by curtailing the size of FES.

Adoption of both the contact-reduction strategies can bring visible reduction in FES confirmed graphically by PMI (Fig 5c and 5d) and BPD (Fig 5e and 5f). In fact, relatively lower cost for both policies could ensure significant suppression of FES in the community. Fig 5c–5f highlight that PMI and BPD can be maximized if the respective cost of any policies becomes lower. This trend gets more exalted when the efficacy of facemasks is relatively high (e.g., the N95 respiratory has 88% efficacy) and individuals in a street maintain a scientifically suggested safe distance (6 feet) [15, 48, 52]. Careful observation of Fig 5c–5f, revealing relative dependability for both policies, apparently similar heat maps can be observed when both costs, efficacy, and distance are preferable. Therefore, respective policy cost and reliability have a profound impact on human decision-making about adopting above mentioned contact-reduction policies.

Finally, ASP provides a good index of how a cost-efficient society is evolved, resulting from individuals’ decision-making at an equilibrium state (Fig 5g and 5h). Ensuring minimum cost for both wearing highly efficacious face coverings and maintaining completely safe social distancing, a cost-efficient public health policy could be designed to control the disease transmission rapidly and effectively in the community. In addition to this, our analysis has also highlighted that mask benefit to others is also required to be ensured as low mask benefit to others (*q* = 0.9) could exacerbate the epidemic scenario.

An insightful point has been unearthed in our analysis. A rigorous comparison of FES, PMI and ASP depicted in Fig 5a(iii), 5c(iii), 5e(iii) and 5g(iii), reveals that when the benefits of mask-wearing by both detected and undetected infectious individuals, contributing to others, are maximized (*q* = 0.1), and strict social distancing is strictly maintained (*d* = 0.9)), then the final epidemic size becomes smaller even in the presence of low mask efficacy (*η* = 0.1). This phenomenon is attributed to the efficacy of source control mechanisms. The rationale behind this lies in the fact that the utilization of masks by infectious individuals can significantly decelerate disease transmission when the maximum benefits of mask-wearing to others are achieved. In contrast, when the benefits of low efficacy (*η* = 0.1) mask wearing by infected individuals, contributing to others is insignificant (*q* = 0.9) in the presence of strict social distancing (*d* = 0.9), the utilization of masks by infectious individuals proves ineffective in decelerating disease transmission. This is evidenced by an increase in the final epidemic size, as depicted in Fig 5b(iii), 5d(iii), 5f(iii) and 5h(iii).

**Fig 6:**
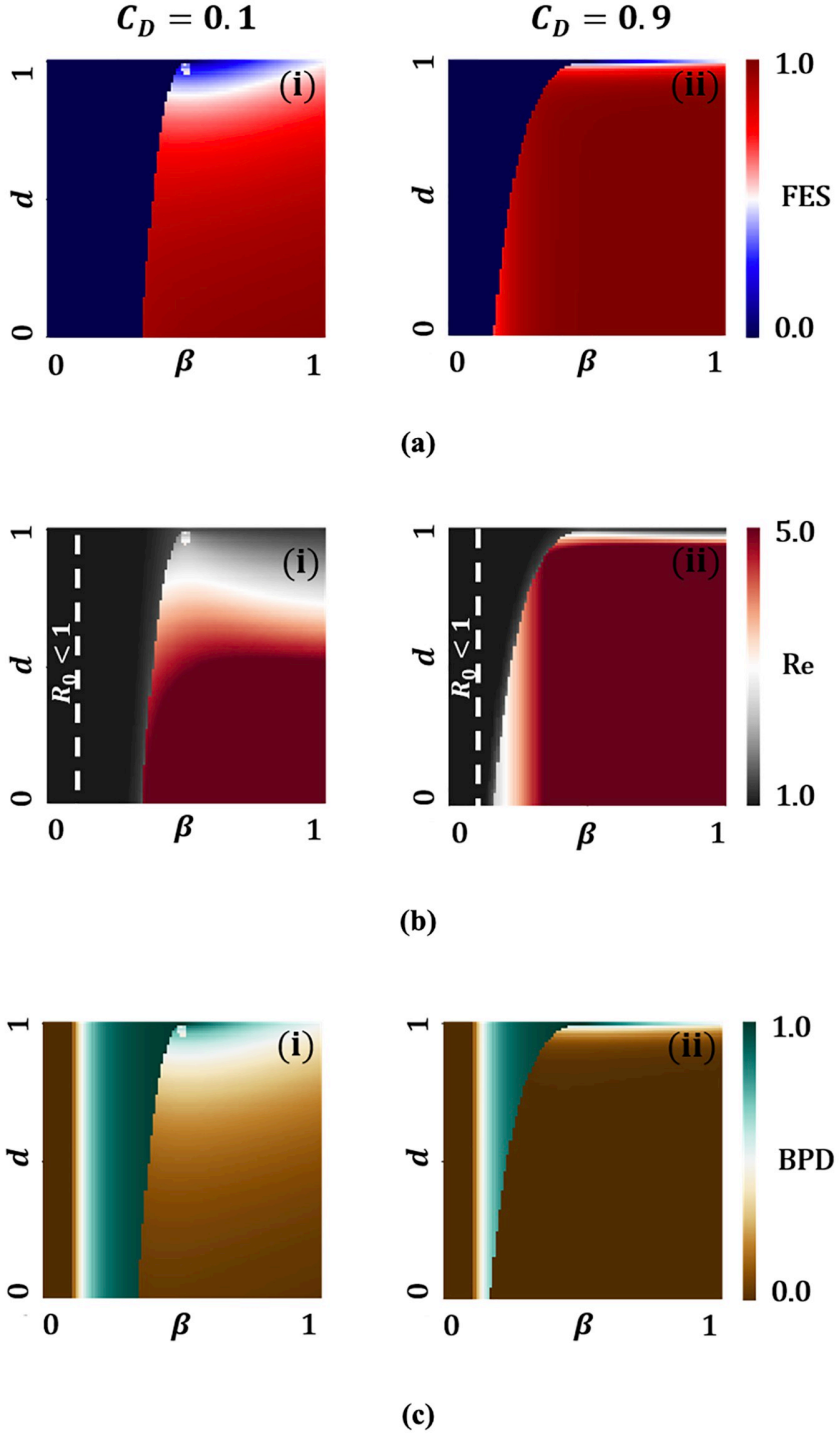
Heat maps of (a) final epidemic size (FES) (b) effective reproduction number ($R_{e}$) and (c) BPD individuals with *C*_*D*_ = 0.1 and 0.9 along *β* and d. All remaining parameters remain constant and are derived from the values listed in the Table 1.

To take into account insightful understanding more broadly of how social distancing work against perceived risk of infection, we display the 2D phase diagram of FES (Fig 6a), $R_{e}$ (Fig 6b), and BPD (Fig 6c) in Fig 6, for the distance maintaining cost *C*_*D*_ = 0.1 and *C*_*D*_ = 0.9, expressed along with *d* versus *β*. As a general tendency, from Fig 6a and 6b, the FES increases as the *β* gets higher and *d* becomes smaller. Another feature that might also affect FES is the relative social distancing cost. Fig 6a(i) highlights that small relative cost (*C*_*D*_ = 0.1) brings reduced FES. This tendency can appear from the influence of BPD (Fig 6c); the implementation of proper social distancing might bring a significant downturn in the FES. However, a careful observation of FES and BPD along with transmission rate *β* reveals that the upsurge of *β* increased the number of infected individuals. In consequence, the strategy of social distancing gets collapsed, which highlights an important drawback of disease control via social distancing, elucidating that people are opting for maintaining safe social distancing when there is an overwhelming risk of getting infected.

Afterwards, Fig 6 illustrates that both numerical and analytic results are complementing each other. Clearly, for *β* ≤ 0.1 and *γ* = 0.1, DFE is evident in the Fig 6a and 6b, as we know from the definition of the basic reproduction number, $R_{0}=\frac{\beta }{\gamma }$. With some inspection, it is not hard to say that $R_{e}\leq R_{0}$ for current epidemic dynamics, $R_{e}$ shows decreased propensity for minimizing *C*_*D*_ and increasing *d*, revealing the important role of social distancing to reduce the infection. Thus, as mentioned earlier, up to a certain level of *β* can avail the benefits of social distancing; however, after the threshold value, the impact of safe social distancing is minimal as this strategy cannot eliminate the disease burden completely from the community.

**Fig 7:**
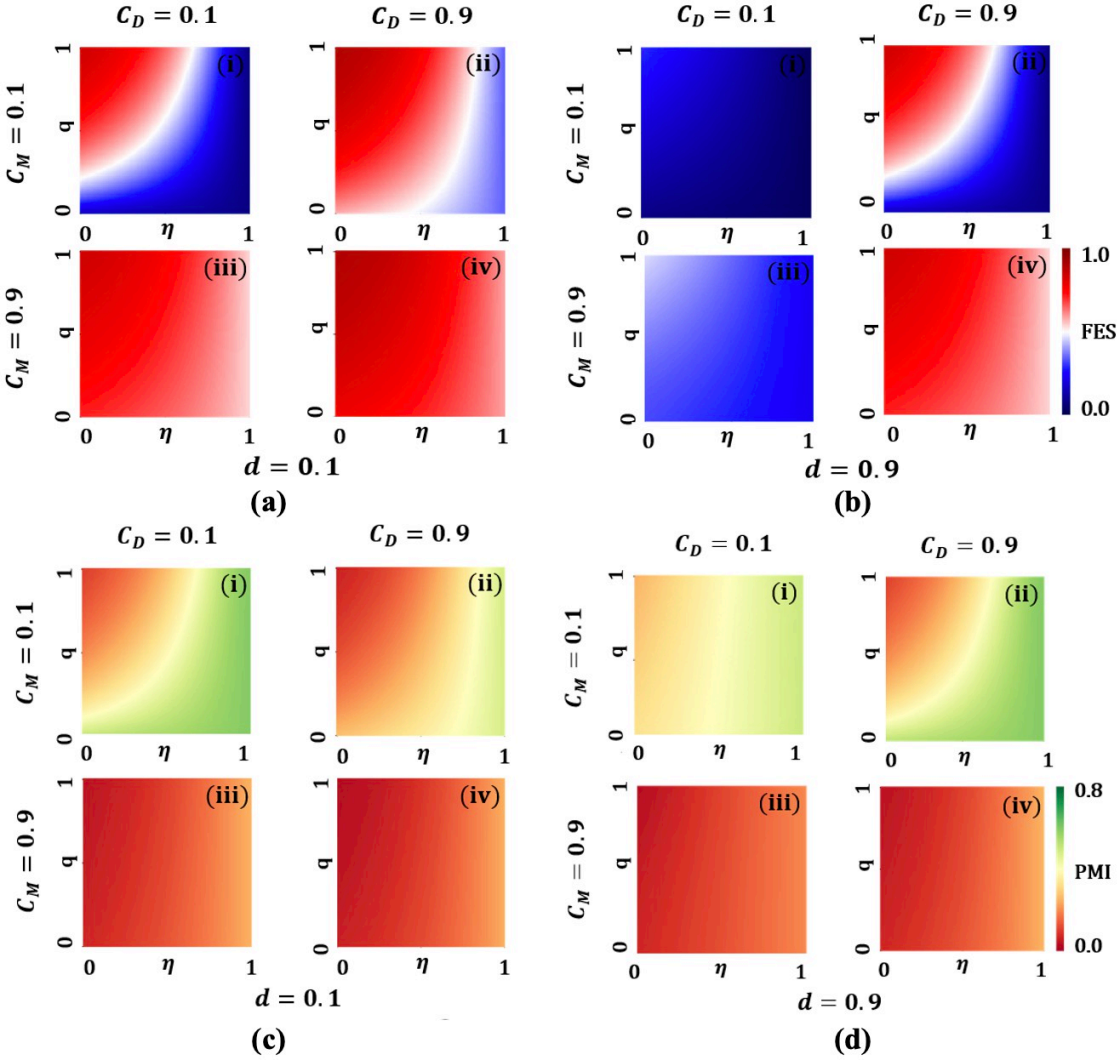
Heat maps of final epidemic size (FES) with *C*_*D*_ = 0.1 and 0.9, *C*_*M*_ = 0.1 and 0.9 along efficacy of face coverings, *η* and mask benefits to others, *q*. Low maintenance of social distancing, *d* = 0.1 is considered for Sub-figure (a), whereas high maintenance of social distancing, *d* = 0.9 is considered for Sub-figure (b). Heat maps of PMI with *C*_*D*_ = 0.1 and 0.9, *C*_*M*_ = 0.1 and 0.9 along efficacy of face coverings, *η* and mask benefits to others, *q*. Low maintenance of social distancing, *d* = 0.1 is considered for Sub-figure (c), whereas high maintenance of social distancing, *d* = 0.9 is considered for Sub-figure (d). All remaining parameters remain constant and are derived from the values listed in the Table 1.

Do facemasks provide protection solely for the wearer, or do they also contribute to the safety of the wearer’s community? Due to this heated discussion, many governments in various countries have mandated and promoted facemasks usage as a contact-reduction measure. Public health experts also recommend effective mask usage to protect both the wearer and those around them from infection. Hence, both undetected and detected infectious people can play a vital role in reducing disease transmission by wearing efficacious face coverings. In this case, people who come in close contact with infectious people could be benefitted, and this ‘benefit to the community level’ is modeled as *q* in our model. Adopting efficacious face coverings by infectious people could be considered as an effective tool of source control. Wearing effective face coverings can reduce the risk of both transmitting and contracting infectious agents, providing mutual protection. The efficacy of a face covering has been modeled as *η* in our framework and can be considered as “protection at the individual level”. To show the interplay between the benefit of wearing a mask to the wearer or others (non-wearer), we portrayed Fig (7a)–(7d) present FES and PMI, respectively.

In the absence of safe social distancing (*d* = 0.1), Fig 7a(i) illustrates that wearing effective face coverings and ensuring maximum benefits to the neighbors of a mask-wearer, FES can be brought down significantly in a low-cost setting. Consequently, Fig 7c(i) illustrates that individuals could be encouraged comprehensively to wear effective face coverings and ensure maximum benefits to the neighbors of a mask-wearer in a low relative economic cost setting. However, from Fig 7c(iv), our results highlight that people could be discouraged to comply with facemasks when facemasks are expensive to buy.

However, in the presence of safe social distancing (*d* = 0.9), Fig 7b presents the same scenario as above. DFE could be achieved in the community, ensuring a low-cost setting for both the contact-reduction measures. Since individuals are more likely to adopt mask-wearing and maintain safe when they are affordable. On the flip side, these contact-reduction measures have no impact in curbing the spread of any airborne disease in exorbitant settings. Similarly, Fig 7d(i) illustrates that people try to get benefitted from safe social distancing in case of low socio-economic cost of maintaining this intervention strategy, which leads to a reduction in the number of masked individuals. Nevertheless, as the cost of maintaining social distancing escalate, efficacious mask usage could be encouraged ensuring affordability.

In summary, during any novel airborne disease outbreak, policymakers immediately think about implementing effective contact-reduction strategies in the community to impede the transmission of the virus. However, the desired success of these intervention policies largely depends on human behavioral responses and decision-making dynamics. Our analysis reveals that when the socio-economic cost of these policies are high, mass people tend to avert adopting mask-wearing and social distancing as effective contact-reduction measures. Policymakers should focus on minimizing relative costs of wearing facemasks and maintaining safe social distancing when deploying these intervention strategies to combat against the disease outbreak. Our analysis highlights that the timely implementation of tailored social distancing could flatten the epidemic curve and serve as an effective epidemic peak delaying strategy, whereas mask-wearing provision can only scale down the severity of epidemic peak. We found that the impact of social distancing amid a disease outbreak can be observed up to a certain level of disease transmissibility. But after that threshold value, social distancing should not be considered as an effective contact-reduction measures. Even though simultaneous adoption of these self-protective measures can significantly curtail disease transmission, only safe social distancing can be prioritized ensuring affordability if there is a shortage of effective face masks. When a country confronts with such crisis of facemasks, policy makers could deploy stringent safe social distancing policy ensuring affordable indirect socio-economic cost and government subsidies. Both undetected and detected infectious individuals can significantly reduce the risk of infection for non-masked individuals by wearing effective facemasks. The choice between emphasizing cost-effective physical distancing or promoting the use of masks during an epidemic outbreak depends on various factors, including the economic situation of a country. Both measures, social distancing and mask-wearing, contribute significantly to reducing the spread of the virus. While masks are commonly considered as a relatively straightforward intervention, it is essential to recognize that their effectiveness can be influenced by factors such as proper usage, mask cost, availability, and public adherence. On the other hand, implementing and encouraging physical distancing measures, such as maintaining a safe distance between individuals, can also be effective in curbing the transmission of infectious diseases. The decision to prioritize one approach over the other may hinge on the economic feasibility and practicality of implementation within a specific country. For instance, in regions facing financial constraints, where the widespread distribution and accessibility of masks may be challenging, emphasis on social distancing could be a more efficient strategy. This is particularly true if implementing and enforcing physical distancing measures can be achieved at a lower cost. It is crucial to acknowledge that the effectiveness of any intervention is context dependent. A comprehensive strategy that considers the local economic conditions, healthcare infrastructure, and public compliance is necessary. Ideally, a combination of both cost-effective physical distancing and mask-wearing, tailored to the specific circumstances of a country, would offer a more robust and adaptable approach in addressing epidemic outbreaks.

Our study analyzes early epidemic-stage human behavior, focusing on non-pharmaceutical interventions like mask wearing and social distancing, excluding vaccination to isolate their effects. While our theoretical model offers several insights, it operates without real-world data, limiting its ability to address the complexity of actual scenarios. Future research endeavors will aim to incorporate epidemic data and employ agent-based modeling techniques.

## Conclusion

To put forth a resilient and sustainable response plan for combating a widespread respiratory disease outbreak, it is imperative to understand the human behavioral mechanism and decision-making dynamics in adopting various intervention strategies. In this study, a novel game-theoretic concept allows us to analyze the interplay of maintaining safe social distancing and efficacious mask-wearing as effective infectious contact-reduction policies under human behavioral dynamics. Our theoretical framework successfully captures most of the salient factors responsible for shaping complex human decision-making dynamics during COVID-19 outbreak. Quantitative analysis of the model reveals that mass people comply with mask-wearing and social distancing when relative inherent costs are affordable for them. Usage of efficacious facemasks by infectious people can significantly limit the probability of virus transmission, which is indeed a prosocial behavior from a public health perspective. Sensitivity analysis of the effective reproduction number ($R_{e}$) quantifies the fact that strict maintenance of social distancing and high efficacy of facemasks are the most desirable factors for achieving prospective success in epidemic control. Policymakers must carefully consider the cost-benefit analysis when implementing social distancing and mask-wearing as infectious contact-reduction measures during an epidemic. Minimizing socio-economic costs associated with these contact-reduction measures can encourage widespread public compliance. In case of unavailability of efficacious facemasks, safe social distancing can be prioritized to forestall the epidemic progression. Social distancing can be an effective epidemic peak-delaying and curve-flattening intervention strategy. However, its impact may diminish in the face of high disease transmissibility. To attain the utmost benefit of safe social distancing, it should be implemented as early as possible. The availability of affordable face coverings and mask-wearing as a source control strategy can significantly reduce the disease burden in society. In the presence of highly virulent respiratory virus variants and when the indirect socio-economic cost of maintaining social distancing is high, our analysis suggests promoting a universal mask policy at a low cost. Finally, countries retain the flexibility to prioritize either strategy, adjusting the stringency of the other based on their socio-economic conditions.

## Supporting information

S1 Text (TXT)
